# Perspective: Examining Conflicts of Interest for Professional Service within the 2020 Dietary Guidelines Advisory Committee

**DOI:** 10.1016/j.advnut.2023.03.009

**Published:** 2023-03-28

**Authors:** Vivica I. Kraak

**Affiliations:** Department of Human Nutrition, Foods, and Exercise, Virginia Polytechnic Institute and State University (Virginia Tech), Blacksburg, VA, United States

**Keywords:** Dietary Guidelines Advisory Committee, conflicts of interest, bias, advisory committee, dietary guidelines

## Abstract

Judgments and integrity uphold professionalism. Failure to manage professional conflicts of interest (COIs) may undermine trust in an individual, practitioner, or institution. This perspective article examines standards for nutrition researchers and practitioners to manage COIs for the Dietary Guidelines for Americans (DGA) process. Thereafter, this article analyzes a study published by Mialon et al. that raised concerns about the expert advisory committee selection process and management of COIs for 20 professionals appointed by the Department of Health and Human Services (HHS) and the USDA, who served on a federal government advisory committee to review evidence for the Dietary Guidelines Advisory Committee (DGAC) 2020 scientific report. The analysis found that Mialon et al. enumerated COIs for each DGAC member with industry, removed from the original context, which prevented readers from assessing the COI risk. Moreover, the USDA ethics office concluded that “the 20 committee members were in full compliance with the federal ethics rules applicable to special government employees.” I conclude that Mialon et al. could use institutional mechanisms to encourage the USDA and HHS to strengthen future COI policies and procedures, aligned with the 2022 National Academies of Sciences, Engineering and Medicine report recommendations to improve the DGA 2025 to 2030 process.

**Statements of Significance**

This is the first article in *Advances in Nutrition* to examine and critique a study published by Mialon et al. in *Public Health Nutrition* in March 2022 that used network analysis to map and enumerate perceived conflicts of interest (COIs) for 20 professionals appointed to serve on the 2020 Dietary Guidelines Advisory Committee. This perspective article synthesizes evidence to clarify the different policies and procedures required by United States government agencies for nutrition professionals to declare significant COIs for research and professional service. This article found that the ethics officer in the Unites States Department of Agriculture (USDA) concluded that “the 20 committee members were in full compliance with the federal ethics rules applicable to special government employees.” Mialon et al. could submit comments to the USDA to strengthen future COI policies and procedures, aligned with the 2022 National Academies report’s recommendations to improve the Dietary Guides for Americans 2025–2030 process.

## Introduction

Judgment and integrity are two features of professionalism [1]. Conflicts of interest (COI) are defined as a conflict between a professional responsibility and personal interest [1]. Strong COI policies, procedures, and management preserve trust in an individual, practitioner, or institution [2]. Failure to acknowledge and manage COIs can erode trust in one’s professional judgment [2].

The standards for evaluating COIs must be interpreted within a specific context and may have different meanings across cultures, countries, institutions, and at different time points [2]. A growing scrutiny of corporate practices has fostered calls to strengthen COI policies to prevent commercial influence on public policy, research, and practice [3,4]. In the United States, concerns have focused on how food and beverage firms influence nutrition research and create research biases that skew funding toward industry benefits [5]. Defining and describing the COI context accurately is essential given the variation across institutions and professional activities [[1], [2], [3], [4], [5]].

This perspective article examines the COI management standards for United States nutrition researchers and practitioners who served as advisory members appointed to the Dietary Guidelines for Americans (DGA) process. The Dietary Guidelines Advisory Committee (DGAC) was appointed by the United States Department of Agriculture (USDA) and Department of Health and Human Services (HHS) agencies to review relevant evidence and write the 2020 scientific report [6]. The DGAC 2020 report was used by the USDA and HHS Secretaries to inform the DGA 2020–2025 report [7]. Thereafter, this article analyzes a study by Mialon et al. [8] published in the United Kingdom’s Nutrition Society journal that raised concerns about the expert advisory committee selection process and management of COIs for 20 DGAC members. This perspective article also examines the consequences of researchers who use social media to make public allegations of professional misconduct of others before conducting due diligence of the Unites States policy context for this issue.

### Disclosing Professional COIs in the United States Context

US researchers and practitioners must properly disclose a significant COI, defined by federal, state, institutional, journal, or other policies [9]. The US HHS Office of Research Integrity (ORI) oversees compliance for the research policies of federal government agencies. The ORI defines a COI as “any financial or other interest that conflicts with the service of the individual because it could significantly impair the individual’s objectivity or could create an unfair competitive advantage for any person or organization” [9]. A COI occurs on a spectrum of activities between cognitive biases (conscious or unconscious), and the COI may result in more serious offenses that lead to professional dishonesty (intentional or unintentional) [1].

The ORI distinguishes between four types of conflicts, including: financial COI, institutional COI, conflict of commitment related to one’s time and effort to a primary employer, and conflict of conscience related to one’s moral beliefs [9]. The ORI has requirements for managing these types of conflicts, such as what needs to be disclosed, how it needs to be disclosed and to whom, and permissible activities [9].

Academic researchers, including faculty, staff, and students, are required to take online COI training every 4 y to improve their understanding of and compliance with COI policies and procedures [9]. Managing a COI means that individuals take appropriate actions to ensure that their personal or financial interests do not adversely influence research and other professional activities [9].

It is important to note that a perceived COI is context-specific and that private employers and government agencies use different COI policies and procedures depending on the individual’s professional role [9]. Institutional COI policies and procedures vary across US government agencies and the activities of more than 1000 federal advisory committees.

The US Office of Government Ethics provides federal agencies with a confidential disclosure form that advisory committee members use to report funding amounts and other COIs. These forms are reviewed by an ethics officer who ensures oversight, accountability, and committee member compliance with specific US policies. The ethics officer may request additional required information from the provisional committee members to ensure that the reporting is accurate before finalizing a committee appointment.

Although the processes for managing COIs are well-established, there are opportunities to increase transparency. Biomedical research maintains publicly accessible registries [10]; however, this practice may not translate effectively for advisory committee service. The US Preventive Services Task Force’s COI policies and procedures used for clinical preventive services could be adapted and tailored for other advisory boards with appointed members who have special interests that may influence their professional judgment about reviewing evidence for recommendations [11].

### Different Types of Bias that May Influence Professional Judgment

Many types of cognitive biases may influence professional judgment and lead to misconduct [1]. A confirmation bias is the tendency to support a belief, evidence, or favor a position consistent with an individual’s values [12]. A confirmation bias may occur by dismissing evidence that contradicts one’s belief or overvaluing evidence that confirms one’s beliefs, regardless of whether the information is factual [12]. Moreover, other types of cognitive biases may influence research processes and outcomes that include reporting, performance, citation, and publication biases [13].

Academic researchers must attract funding to support their research to be successful in their professional careers. A researcher who has not adequately disclosed or managed a significant COI, or whose professional judgment was influenced by a cognitive bias, may experience many consequences. Non-compliance with the appropriate management of financial or institutional COI policies, and professional misconduct, could diminish public trust and peer respect, cause suspension or termination of sponsored research, and produce reputational damage related to disciplinary actions taken by an employer and professional society for breaching a code of conduct or institutional policies [2,[9], [10], [11], [12], [13]].

Receiving funds from a food, beverage, restaurant, or pharmaceutical firm must be interpreted within the context of the study purpose and design, research objectives, funding received, and how the results are described in a peer-reviewed publication. Therefore, international researchers who study governance, ethics, corporate influence, and COIs in public health [14] must conduct adequate due diligence to ensure that they interpret a country’s policy context accurately.

Medical, nutrition, and public health practitioners could take proactive actions by adhering to the ethical codes of conduct required by professional societies to maintain their credentials [[15], [16], [17], [18]]. Government agencies and academic settings could also strengthen institutional COI policies and procedures and enhance transparency to protect the policymaking process from undue commercial influence that may undermine the diets and health of populations [[1], [2], [3], [4], [5]].

### The DGA Process, 1990–2020

The process to develop the DGA has evolved over 30 y. The DGA process is mandated by a federal 1990 law that requires the USDA and HHS Secretaries to appoint an expert advisory group every 5 y to review the science and update the national dietary guidelines used to inform food and nutrition policies and programs [19]. The USDA and HHS appointed the DGAC members, many who were nominated by their peers, who had served their professional communities, and who had published on topics prioritized by the USDA and HHS agencies [19]. The DGAC 2020 members evaluated the scientific evidence for *a priori* research questions about diet and health outcomes that were selected by the 2 federal agencies, not the members [19].

**FIGURE 1:**
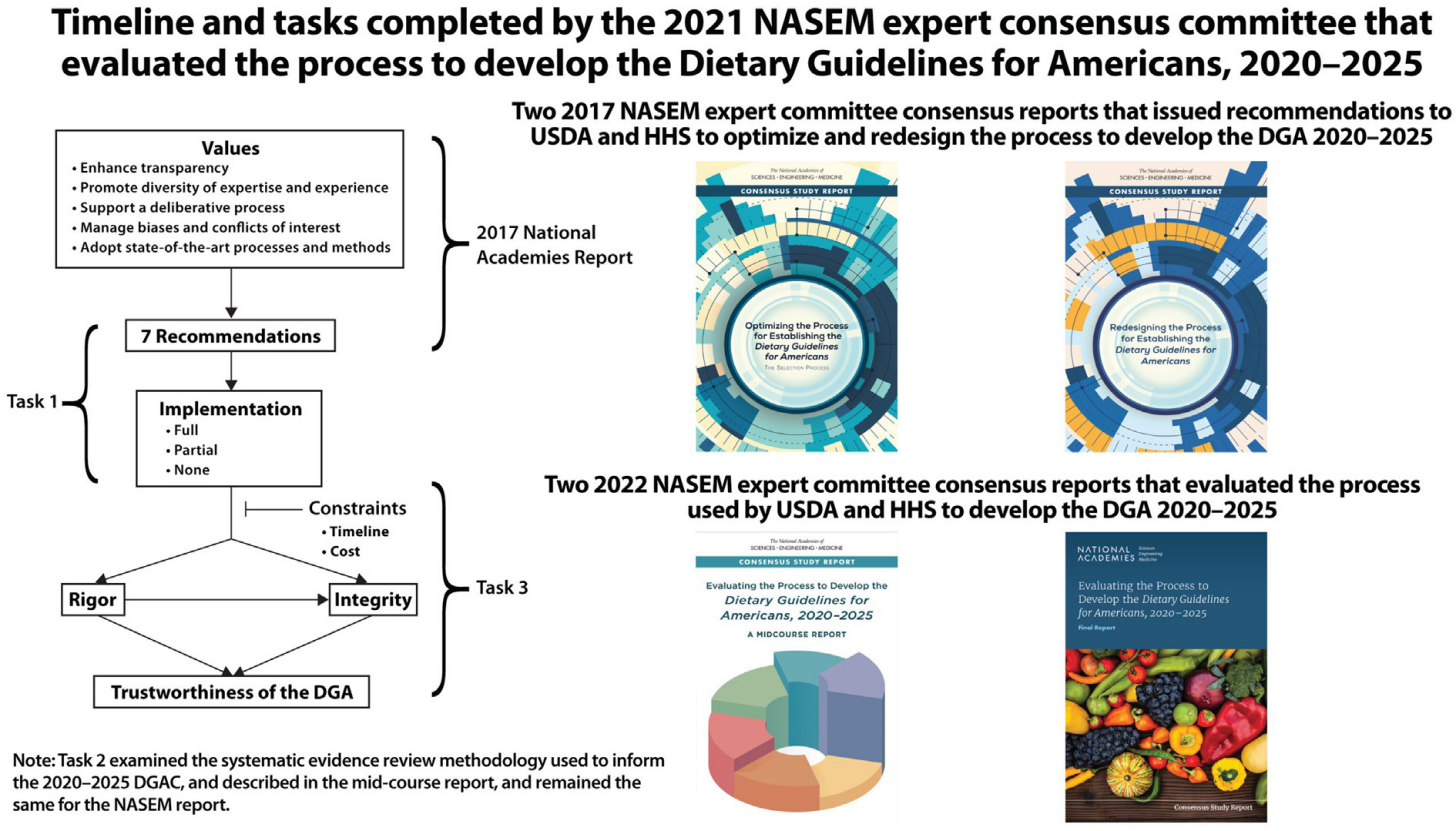
Timeline and tasks completed by the 2021 NASEM expert consensus committee that evaluated the process to develop the DGA, 2020–2025. DGA, Dietary Guidelines for Americans; DGAC, Dietary Guidelines Advisory Committee; HHS, Health and Human Services; NASEM, National Academies of Science, Engineering, and Medicine. Figure was adapted and reproduced with permission from the National Academy of Sciences, Courtesy of the National Academies Press, Washington, DC.

Public concerns led to the United States Congress to appropriate funds in 2016 to direct the USDA Secretary to engage National Academies of Sciences, Engineering, and Medicine (NASEM), an independent organization that advises the federal government and US Congress on science, health, and medicine, to conduct a study of the DGA process [20]. NASEM released 2 consensus committee reports in 2017 that provided recommendations for HHS and USDA to optimize the DGA selection process [21] and redesign the DGA process [22] (Figure 1). The 2017 NASEM reports aimed to improve the scientific integrity of the DGA process to enhance transparency, manage bias and COI, promote diverse expertise, support a deliberative process, and adopt state-of-the-art methods [20,21]. However, the 2020 DGAC was appointed before USDA and HHS had fully implemented the NASEM report recommendations.

In 2021, the US Congress Appropriations Act mandated a comprehensive review of the entire DGA process by an *ad hoc* 12-member NASEM expert committee charged with three tasks (Figure 1). Task 1 determined the extent (that is, full, partial, or no implementation) that the USDA and HHS had implemented the 2017 NASEM reports’ recommendations. Task 2 examined the and systematic review criteria and process used to inform the 2020 to 2025 DGA to ensure that the evidence was current, rigorous, generalizable, and applicable to public health nutrition guidance. Task 3 evaluated the process compared with the full implementation of the seven 2017 NASEM recommendations regarding the timeline, cost, and integrity of the 2020 implemented guidelines.

The mid-course NASEM 2022 report concluded that USDA and HHS needed to take additional steps to fully implement the 2017 NASEM recommendations to prepare for the DGA 2025 to 2030 process [23]. The final NASEM 2022 report concluded that the “management of COI of individual members of the DGAC committee was updated after the 2017 NASEM report, and the management of risk of bias as part of the systematic evidence review process was aligned with practices of other leading systematic review organizations; however, the USDA and HHS have opportunities to better assess and manage relevant COI” in the future [23].

The USDA and HHS selection and committee process for the DGAC 2020 were documented as required by the 1972 Federal Advisory Committee Act [24]. The DGAC committee members followed the USDA and HHS institutional COI policy and procedures, as described in the DGAC 2020 report (7 Page 52). Each member completed the confidential financial disclosure Form 450 [25] issued by the US Office of Government Ethics to report funding and COI within the past 12 months before the committee service. This is a crucial point because the Mialon et al. [8] study described below examined public records to identify industry activities for each DGAC member over 20 years, despite the US government requirement to report for the past 12 months.

The USDA ethics officer’s review concluded that “none of the 20 committee members reported any entries on their form that would prevent them from being appointed and that they were in full compliance with the federal ethics rules applicable to special government employees (7 Page 52).” In 2018, the USDA responded to the US Congress to explain that the two federal agencies had considered but decided not to publicly post confidential information about the provisional DGAC appointees because of privacy concerns [26,27].

Details of the DGA processes are described elsewhere [18,22,[28], [29], [30], [31]]. The 835-page DGAC 2020 scientific report [7] was shared with the USDA and HHS Secretaries for internal institutional review, and the recommendations were translated into simple messages before the agencies released the 164-page DGA 2020 to 2025 report in December 2020 [8].

### Critique of a Study that Examined COIs for the 2020 DGAC Members

A study by Mialon et al. [8] examined the incidence and prevalence of industry-related COIs for 20 expert advisory committee members appointed by the USDA and HHS Secretaries to produce the DGAC 2020 scientific report. The authors defined COI as “relationships between DGAC members and an industry actor in a given year” [8]. No other details were provided to define industry actors, establish funding thresholds, or designate the timeframe used in their analysis. The authors did not describe specific research questions or objectives in the methods [8]. It was unclear why the study had examined individual COI for the 20 DGAC members but not the institutional COI policies and procedures used by the USDA and HHS to select the committee.

The Mialon et al. [8] study reported searching the Web of Science for COI evidence from the DGAC members’ institutional affiliations, funding acknowledgments, and declaration of interests in their publications [8]. Best practices for reviews suggest that ≥2 researchers independently extract information but were not described in Mialon’s article. Network analysis and data visualization were used to map each committee member’s relationship with industry, removed from the original context, which prevented readers from assessing the COI risk. The authors did not publish supplemental evidence tables for independent verification.

Mialon et al. [8] listed the frequency of type of COI for all DGAC members in a table that totaled 714 COI, with the highest for research funding (*n* = 289 COI), board member (*n* = 155 COI), and consultant (*n* = 105 COI). This study treated each COI equally and provided no detailed information about the honorarium amount received for consultant services, whether the board membership was active, or whether the DGAC member received any monetary compensation for the scientific or professional board membership.

A second table listed the names of each of the 20 DGAC members by COI that ranged from 0 to 152, and the number of industry actor connections for each member that ranged from 0 to 31 [6]. Mialon et al. [8] reported that “95% of the 20 committee members had a COI with the food, and/or pharmaceutical industries including: Kellogg, Abbott, Kraft, Mead Johnson, General Mills, Dannon, and the International Life Sciences Institute and that “research funding and advisory committee or executive board members accounted for >60% of the total COI documented” [8]. These authors concluded that “widespread COI of the DGAC members did not meet the recommended institutional standard of transparency to make the COI publicly available” [8]. However, the Mialon et al. [8] study was not designed to examine the institutional context in which the DGAC members were engaged, which would have revealed that the NASEM standards for transparency and scientific integrity were met.

Conducting research motivated by a desire to pursue righteous ends is known as “white hat” bias that may result in misleading information [32]. Therefore, authors should declare their own financial support received from private foundations or non-profit organizations. Mialon et al. [8] identified their study sponsor as the Nutrition Coalition, and the executive director was the fourth author. Yet, the article declared “no input” while the executive director of the sponsoring agency contributes as an author is a direct failure to meet the evidentiary standards for scientific integrity. The study also failed to declare any of the authors’ other funding sources, professional COIs, and did not publish supplemental evidence tables for independent review.

### Perils of Promoting Misleading Study Findings on Social Media

In the digital age, there are consequences when individuals allege that health or nutrition professionals have not reported a significant COI that was properly managed by an institutional procedure at a specific point in time. This is especially problematic when individuals promote misleading findings on social media to the public with limited details of a policy context.

Obsessive criticism occurs when individuals attack scientists for the content and conclusions of a scientific report or attack a person’s character rather than discussing the evidence used to support conclusions in a study or report [33]. The COI definition used by Mialon et al. [8] represented a “politics of objectivity with allegations that COI is used to undermine others’ credibility or participation” [34]. Moreover, these researchers engaged in digital vigilantism [35] to seek public justice using social media to attack the DGAC professionals who produced the scientific report [6] used to inform the DGA 2020 to 2025 report [7].

Obsessive criticism and digital vigilantism have been used to target other expert advisory bodies. For example, a freelance journalist published an article in the 2015 *BMJ* titled, *Sugar: spinning a web of influence* [36] presented as a social network analysis study. The article used an infographic to show the food, beverage, and pharmaceutical industry ties to nutrition researchers who had served on the United Kingdom's Scientific Advisory Committee on Nutrition. The journalist alleged that certain committee members at the University of Cambridge had industry ties and interactions that influenced their scientific judgment [36]. Several colleagues replied to the *BMJ* editor to support the advisory members by providing the context for, and benefits of, the industry interactions [[37], [38], [39]]

Context is key when conducting COI research to determine whether and how public health professionals should interact with food and beverage industry representatives [40]. The US nutrition research community and professional societies have updated guiding principles and frameworks to improve transparency, scientific integrity, and trust and to manage COIs for food and nutrition research that involves and is funded by industry actors [[41], [42], [43]].

European journal editors suggest disclosure guidelines for authors when submitting manuscripts for peer review that distinguish among *1*) studies financed by industry (in part or total) with a clear declaration that the industry was not involved in the study design, execution, analysis, or interpretation; *2*) studies sponsored by industry (in part or total) with a clear declaration of which steps involved industry; and *3*) studies funded and conducted by industry with no external partners [44].

Publishing information that harms an individual’s reputation without evidence is called defamation. Mialon and colleagues targeted and defamed 20 unpaid volunteer scientists who donated their professional time and expertise to the US government, which demonstrated poor professional judgment and integrity. The United Kingdom Nutrition Society leadership and editors for *Public Health Nutrition* could maintain the record for scientific integrity by requesting that Mialon and colleagues publish a timely correction, and also write a public letter of apology for the reputational harm to the 20 DGAC members caused by the study. Mialon et al. [8] could also submit public comments to the USDA and HHS to strengthen institutional COI policies and procedures, aligned with the 2022 NASEM report recommendations to improve the DGA 2025–2030 process [23,24].
